# Lufenuron induces reproductive toxicity and genotoxic effects in pregnant albino rats and their fetuses

**DOI:** 10.1038/s41598-020-76638-6

**Published:** 2020-11-11

**Authors:** Wesam T. Basal, Abdel Rahman T. Ahmed, Aya A. Mahmoud, Amel R. Omar

**Affiliations:** Department of Zoology, Faculty of Science, Cairo University, Cairo, Egypt

**Keywords:** Developmental biology, Molecular biology, Zoology

## Abstract

Insecticides and other agrochemicals have become indispensable components of the agricultural system to ensure a notable increase in crop yield and food production. As a natural consequence, chemical residues result in significantly increased contamination of both terrestrial and aquatic ecosystems. The present study evaluated the teratogenic, genotoxic, and oxidative stress effects of residual-level lufenuron exposure on pregnant rats during the organogenesis gestational period of both mother and fetus. The tested dams were divided into three groups; control (untreated), low-dose group (orally administered with 0.4 mg/kg lufenuron) and high-dose group (orally administered with 0.8 mg/kg lufenuron). The dams of the two treatment groups showed teratogenic abnormalities represented by the asymmetrical distribution of fetuses in both uterine horns, accompanied by observed resorption sites and intensive bleeding in the uterine horns, whereas their fetuses suffered from growth retardation, morphologic malformations, and skeletal deformations. Histologic examination of the liver and kidney tissues obtained from mothers and fetuses after lufenuron exposure revealed multiple histopathologic changes. DNA fragmentation and cell cycle perturbation were also detected in the liver cells of lufenuron-treated pregnant dams and their fetuses through comet assay and flow cytometry, respectively. Moreover, lufenuron-induced oxidative stress in the liver of mothers and fetuses was confirmed by the increased malondialdehyde levels and decreased levels of enzymatic antioxidants (glutathione peroxidase and superoxide dismutase). Taken together, it can be concluded that lufenuron has a great potential in exerting teratogenic, genotoxic, and oxidative stresses on pregnant rats and their fetuses upon chronic exposure to residual levels during the organogenesis gestational period. The obtained results in the present study imply that women and their fetuses may have the same risk.

## Introduction

The growing world economy and aggressive competition in the food industry have inevitably forced insecticides and agrochemicals to become an indispensable component of the agricultural system to ensure that crop yields and food production are increased^1^. Unfortunately, these competitions have totally focused on increasing production, intentionally ignoring the impact of chemicals on environmental and human health^2^.

The widespread accumulation of agrochemical residues during the last few decades, especially insecticides, has resulted in significantly increased contamination of terrestrial and aquatic ecosystems, poisoning human food and the environment^3^. Residues of pesticides were detected in several types of everyday food, water, and animal food^4,5^. Quite recently, considerable attention has been paid to the presence of pesticide residues that exceed the maximum limit in several crops in Egypt. Moreover, the presence of unregistered pesticides was also reported^6–9^.

Agricultural workers are at a higher risk of exposure to the adverse effects of insecticides. The general population may be also affected either by consuming contaminated food and water or prolonged continued exposure to sublethal doses of pesticides^10–12^. Toxaphene, DDT, and endosulfan were reported to be bioaccumulated in soil, aquatic sediments, and aquatic food chains which are eventually returned to humans^13–15^. Compounds, such as hexacyclohexanes (HCH) and chlordane, are volatilized after field application, transported by atmospheric processes, condensed in cooler climates, and distributed to regions far away from the application areas^16,17^.

Women, children, and even developing fetuses are not spared, since they are unintentionally and, most of the time, unknowingly exposed to lethal and sublethal doses of insecticides. Maternal environmental exposure to chemical pollutants was recently ranked as the second most important cause of infant mortality in developing countries^18–20^. Several studies have documented the possible association between parental exposure to pesticides and congenital anomalies^21–23^. Pesticides that readily penetrate the placental barrier have been detected in amniotic fluid, umbilical cord blood, meconium, and infant urine, indicating the exposure of human fetuses to pesticides^24–26^.

Evidence has accumulated over the last two decades, suggesting that several pesticides induce genetic damage to humans, domestic animals, and economy-impacting plants^27^. It has also been established that exposure to chemicals that induce mutations is a main contributor to the development of several human cancers^28,29^. As a valuable tool in this field, the comet assay is considered as one of the most promising methods for genotoxicity studies against environmental chemicals. The alkaline comet assay (single-cell gel electrophoresis) is the most widely used method for measuring DNA damage in eukaryotic cells^30^. This assay is a rapid, sensitive, and inexpensive test for detecting DNA damage, which is widely used for evaluating the genotoxicity of chemical compounds under laboratory and field conditions in mice^31^, zebra fish^32^, human germ cells^33^, and *Drosophila melanogaster*^34^.

Cell cycle arrest is initiated by DNA damage or cell cycle failure. When the DNA damage is severe or the cell cycle failure is irreversible, the cell progresses to apoptosis. Treatment with certain DNA-damaging agents arrests cell cycle progression by activating phase-specific checkpoints^35^ or inducing cells to commit suicide through apoptosis^36^. Different cytotoxic agents, radiation, or drug-induced cell death can induce G2/M phase accumulation^37,38^. Cell cycle analysis showed G2/M phase arrest and apoptosis in human erythroleukemia (K562) and human breast cancer (MCF-7) cell lines after exposure to aniline THDA and morphine, respectively^37,39^. Fortunately, in the second half of the twentieth century, several flow cytometry assays have been developed to analyze the cell cycle, and the simplest of these methods depend on a single-time point cell measurement^40,41^.

Exposure to insecticides also induces biochemical alterations, including oxidative stress and lipid peroxidation. However, in the context of the developmental origin of health and disease, the putative trans-generational effects of pesticide exposure are insufficiently studied^42^. The effects of pesticide exposure on humans in the gestational period is seldom studied; however, there is increasing evidence on the role of various exposures during early life (prenatal and post-natal) on adult metabolism and biochemical status^43^.

Lufenuron is an ISO-approved efficient acylurea insect growth regulator (IGR) that was classified safe^44^. However, Salokhe et al.^45^ concluded that exposure to a very low dose of lufenuron may lead to teratogenic effect in vertebrates, whereas Eid et al.^34^ have pointed out its possible mutagenic and genotoxic effects on *D. melanogaster*. This study assumes that lufenuron may be able to cross the placenta and has teratogenic and genotoxic effects on rat fetuses and aims to evaluate the teratogenic, genotoxic, and oxidative stress effects on both pregnant rats and their fetuses upon exposure to residual levels of lufenuron during the organogenesis gestational period.

## Results

### Assessment of reproductive teratogenicity of lufenuron

Pregnant albino rats of the three groups (control group, low-dose group; LD, and high-dose group; HD) did not show any obvious sign of toxicity or abnormal behavior. Neither mortality nor abortion was recorded among the dams of any of the groups throughout the experiment. The average maternal body weight that was recorded for the three tested groups throughout the gestational period demonstrated a significant reduction in weight gain during the 3 weeks of gestation in HD group (48.4 ± 1.99) when compared to LD group (60.2 ± 1.74) and control group (77.30 ± 2.07) (Table 1).

**Table 1:** Reproductive parameters of pregnant rats treated with two doses of lufenuron. Data are presented as means ± standard errors.

Parameter/group	Control	Low dose	High dose
Body weight gain (g)	77.30 ± 2.07	60.2 ± 1.74*	48.4 ± 1.99*^,#^
Uterus weight (g)	43.50 ± 0.75	29.07 ± 0.79*	23.74 ± 0.76*^,#^
Total implantation sites	10.6 ± 0.28	9.2 ± 0.22	9.7 ± 0.27
Resorption (%)	0.00 ± 0.00	18.27 ± 1.85*	51.81 ± 1.63*^,#^
Post-implantation loss index (%)	0.00 ± 0.00	31.22 ± 1.73*	62.77 ± 2.36*^,#^

*Significant difference (*P* < 0.05) as compared to the control.

^#^Significant difference (*P* < 0.05) as compared to the low dose.

**Figure 1:**
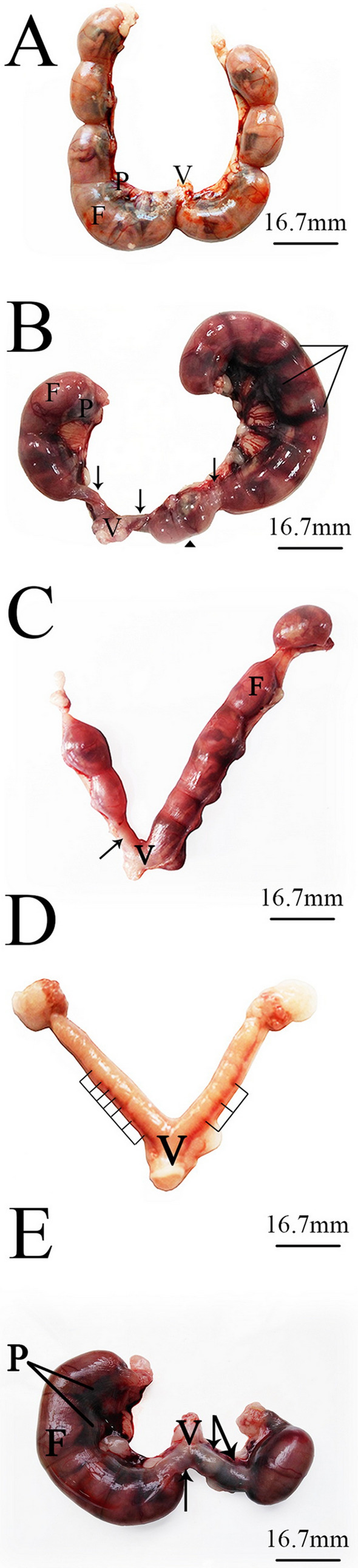
Images of the uterus of pregnant albino rats removed on the 20th day of gestation. (**A**) Control group: the uterus showed normal distribution of fetuses in the two horns. (**B**,**C**) Low-dose group: asymmetrical distribution of fetuses in the two horns with notable resorption sites (arrow). A dead fetus (head arrow) and uterine bleeding (lines) were also detected at the left horn. (**D**,**E**) High-dose group: complete resorption was recorded in several cases (**D**), and clear resorption sites and intensive bleeding in the uterine horns in others (**E**). *F = fetus, P = placenta, V = vagina.

Uteri obtained from pregnant dams via cesarean section operations were weighed and carefully examined for abnormalities. Those obtained from the dams in the control group revealed a normal distribution of implanted fetuses between the two horns (Fig. 1A). Uteri of the dams in the LD group showed asymmetrical distribution of fetuses in the two horns, accompanied by multiple resorption sites (Figs. 1B,C). Bleeding was observed in several cases in the uterine horns of dams in the HD group (Fig. 1D), which was consistent with the notable early and complete resorption sites (Fig. 1E). A significant reduction in the recorded uterine weights of dams from both treatment groups, compared with the control group, was observed. No significant changes in the total number of implantation sites was revealed in both treated groups. The percentage of resorption was significant in HD (51.81 ± 5.61) compared to LD (18.27 ± 5.85) and control (0.00 ± 0.00). There was a significant increase in the percentage post-implantation loss index in HD (11.21 ± 1.21 and 62.77 ± 2.36, respectively) when compared to LD (7.04 ± 1.40 and 31.22 ± 5.46, respectively) and control (0.00 ± 0.00) (Table 1).

### Morphologic examination of fetuses

**Figure 2:**
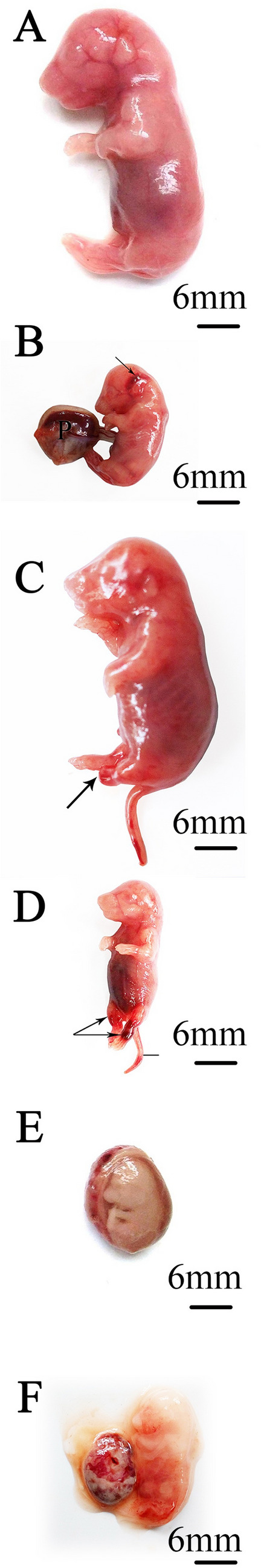
Images of fetuses on the 20th day of gestation. (**A**) Control group: fetuses showed normal morphology and length. (**B**) Low-dose group: fetuses suffered from head hematoma. (**C**,**D**) High-dose group: fetuses suffered from total foot loss (arrow), (**C**) with leg (arrows, **D**) and tail (line, **D**) hematoma. (**E**,**F**) Dead fetuses retrieved from dams of both treatment groups. *P = placenta.

Maternal exposure to the insecticide resulted in notable growth retardation of fetuses, as implied by the significantly reduced number of live fetuses, as well as their weights and lengths (*P* < 0.05) that were observed in both treatment groups, compared to those of fetuses in the control group. Hematoma was recorded to occur more frequently in the HD group, compared with the LD and control groups (Table 2). All of the measured morphologic parameters (shape, weight, and length) for the fetuses retrieved from dams in the control appeared normal (Fig. 2A). On another note, the fetuses extracted from lufenuron-treated dams showed visible dead cases and multiple morphologic malformations; including hematoma, total foot loss (Fig. 2B–F).

**Table 2:** Morphological examination of the fetuses. Data are presented as means ± standard errors.

Parameter/group	Control	Low dose	High dose
Fetal weight (g)	2.27 ± 0.01	1.77 ± 0.02*	1.73 ± 0.12*
Fetal length (cm)	5.68 ± 0.01	4.90 ± 0.02*	4.92 ± 0.12*
Alive	10.60 ± 0.28	6.60 ± 0.15*	3.10 ± 0.09*^,#^
Dead	0.00 ± 0.00	0.90 ± 0.16*	1.2 ± 0.18*^,#^
Hematoma	0.00 ± 0.00	1.70 ± 0.14*	2.5 ± 0.15*^,#^

*Significant difference (*P* < 0.05) as compared to the control group.

^#^Significant difference (*P* < 0.05) as compared to the low dose group.

### Staining for skeletal variations and malformations

**Figure 3:**
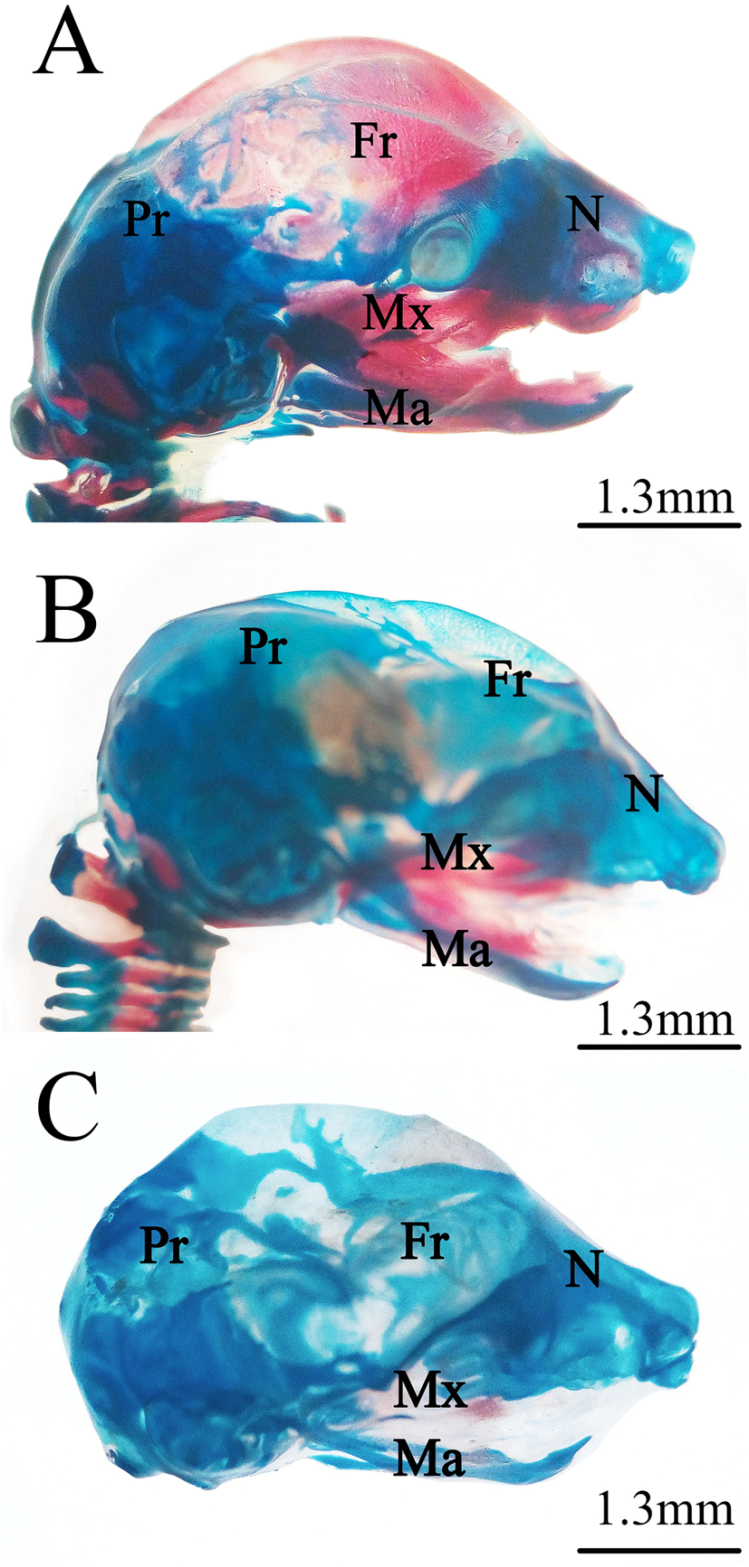
Photomicrographs of the cranial skeleton of fetuses on the 20th day of gestation stained using the Alcian blue–Alizarin red double staining method. (**A**) Control group: normal ossification of the cranial bones. (**B**) Low-dose group: incompletely ossified cranial bones. (**C**) High-dose group: unossified cranial bones. *N = nasal, Mx = maxilla, Ma = mandible, Fr = frontal, and Pr = parietal.

**Figure 4:**
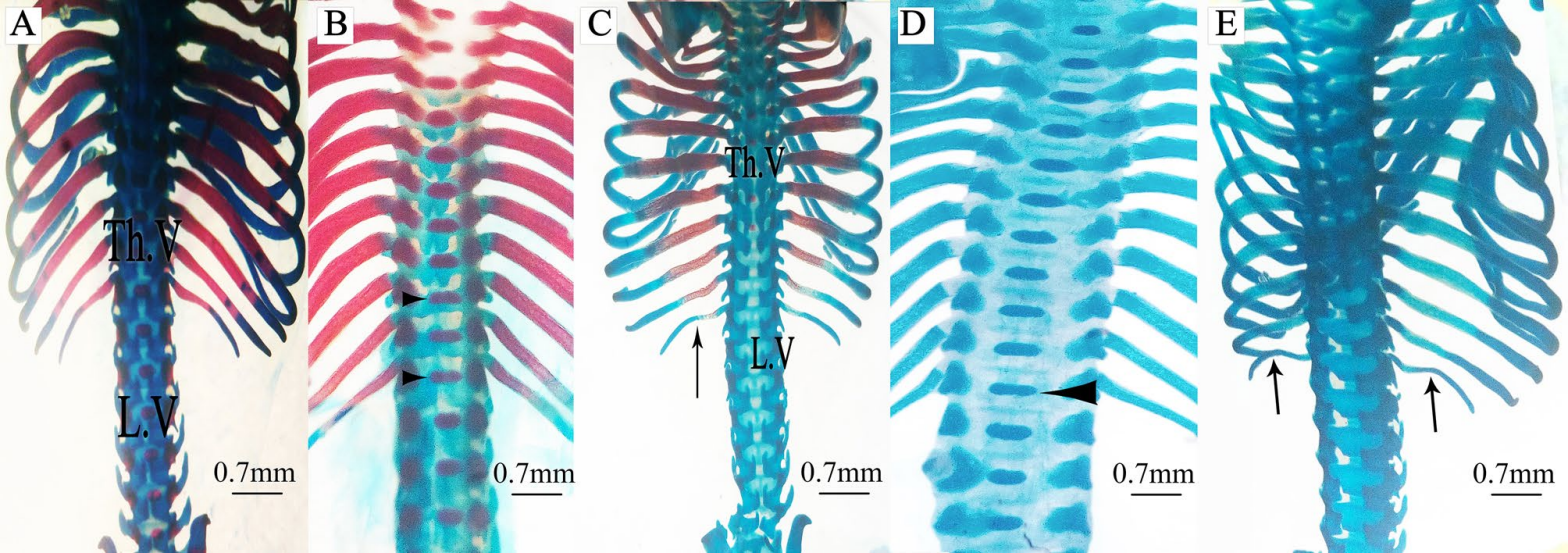
Photomicrographs of the vertebral column and ribs of fetuses on the 20th day of gestation stained using the Alcian blue–Alizarin red double staining method. (**A**) Control group: vertebral column showed complete ossification of all vertebrae and normal appearance of ribs. (**B**,**C**) Low-dose group: vertebral column showed dumbbell-shaped thoracic vertebrae (head arrow), incomplete ossification of the last rib (arrow), and unossified lumbar vertebrae (LV). (**D**,**E**) High-dose group: vertebral column showed dumbbell-shaped thoracic vertebrae (head arrow), unossified thoracic and lumbar vertebral centra, and unossified curved last rib (arrow, e). *Th.V = thoracic vertebra and LV = lumbar vertebra.

**Figure 5:**
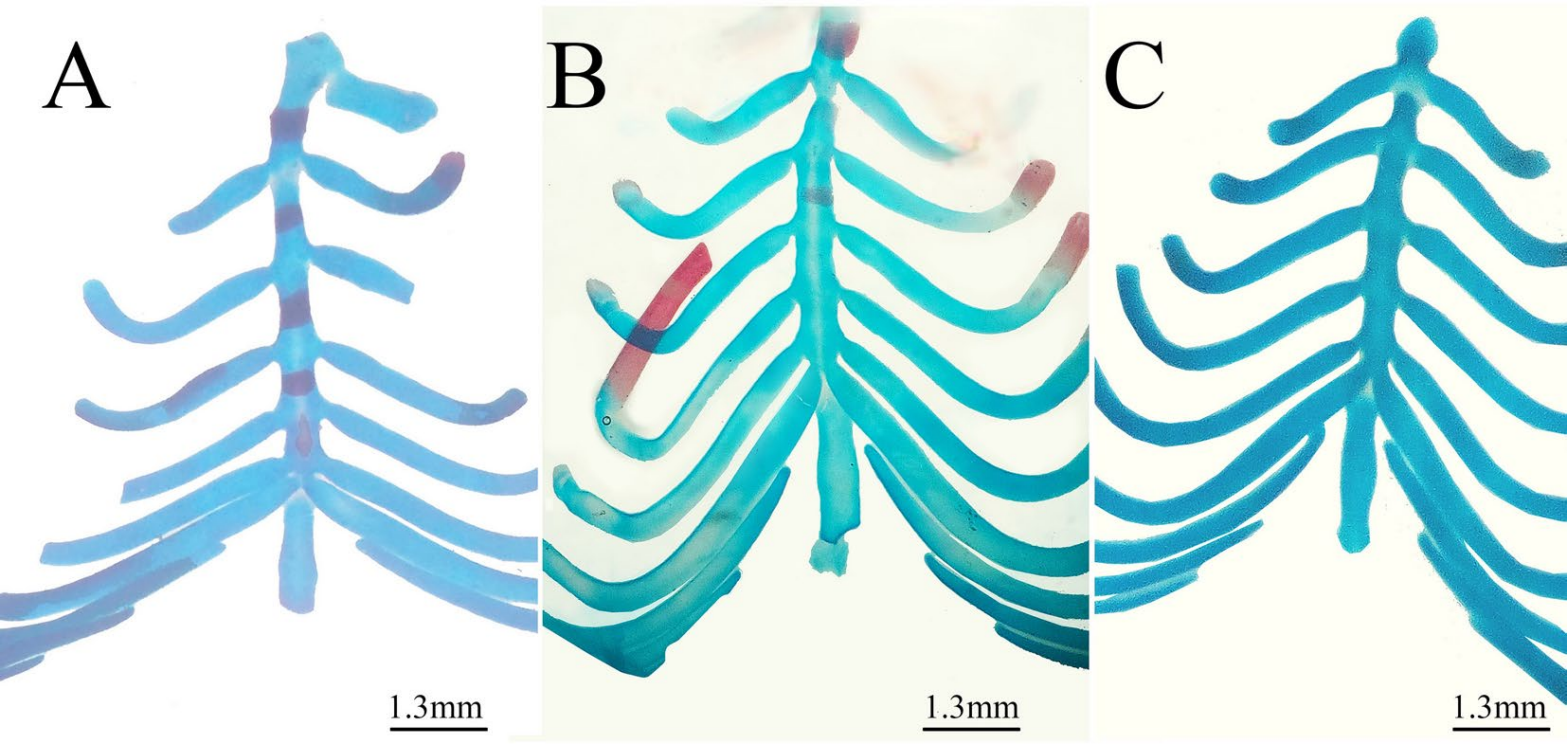
Photomicrographs of the sternum of fetuses on the 20th day of gestation stained using the Alcian blue–Alizarin red double staining method. (**A**) Control group: ossification of the sternum. (**B**) Low-dose group: incomplete ossification of the sternum. (**C**) High-dose group: unossified sternbrae.

**Figure 6:**
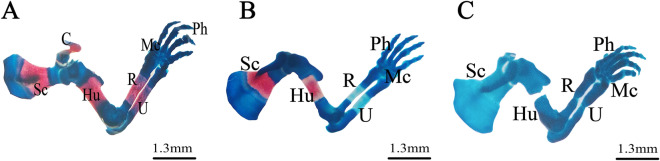
Photomicrographs of the pectoral girdle and forelimb of fetuses on the 20th day of gestation stained using the Alcian blue–Alizarin red double staining method. (**A**) Control group: pectoral girdle and forelimbs showed complete ossification of all bones. (**B**) Low-dose group: lack of ossification of radius, ulna, and metacarpal bones. (**C**) High-dose group: incomplete ossification of all bones and abnormally shaped radius and ulna. *Sc = scapula, Hu = humerus, R = radius, U = ulna, Mc = metacarpals, and Ph = phalanges.

**Figure 7:**
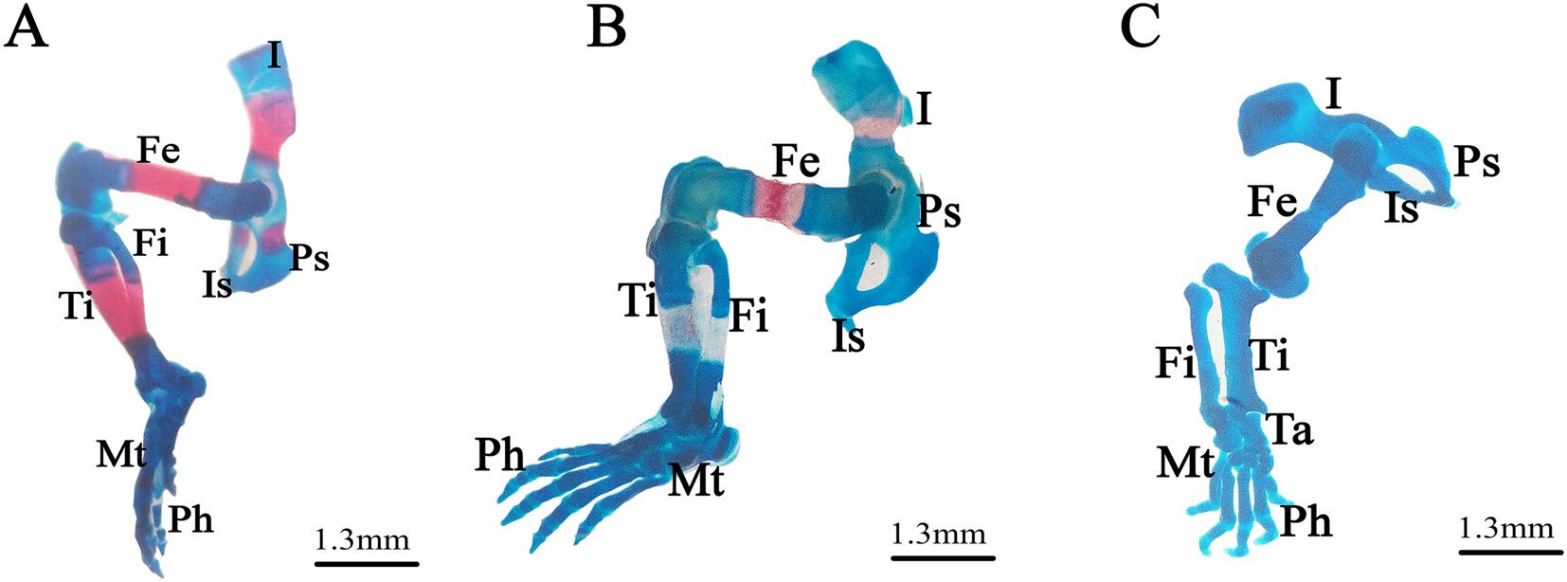
Photomicrographs of the pelvic girdle and hind limbs of fetuses on the 20th day of gestation stained using the Alcian blue–Alizarin red double staining method. (**A**) Control group: pelvic girdle and hind limbs showed ossification of all bones. (**B**) Low-dose group: incomplete ossification of the pelvic girdle and unossified hind limb bones (tibia, fibula, metatarsals, and phalanges). (**C**) High-dose group: completely unossified bones. *I = ilium, Is = ischium, Ps = pubis, Fe = femur, Fi = fibula, Ti = tibia, Mt = metatarsals, and Ph = phalanges.

Examination of Alcian blue–Alizarin red stained fetuses revealed several skeletal ossification delay and abnormalities in the groups exposed to different lufenuron concentrations during the organogenesis gestational period (Table 3, Figs. 3, 4, 5, 6, 7).

**Table 3:** (A) Skeletal ossification of rat fetuses on 20th day of pregnancy. (B) Skeletal deformation of rat fetuses on 20th day of pregnancy.

Skeletal area	Ossification status	Control	Low dose	High dose	Fisher exact test		
		Number (%)	Number (%)	Number (%)	Value^1^	Value^2^	Value^3^
(A)							
Frontal bone	Complete	30 (100)	18 (60)	9 (30)			
	Unossified	0 (0)	12 (40)	21 (70)	0.0001	0.00001	0.037
Parietal bone	Complete	19 (63.4)	6 (20)	6 (20)			
	Unossified	11 (36.6)	24 (80)	24 (80)	0.0014	0.0014	*1*
Thoracic vertebrae	Complete	30 (100)	13 (43.3)	5 (16.7)			
	Unossified	0 (0)	17 (56.7)	25 (83.3)	0.00001	0.00001	0.047
Lumbar vertebrae	Complete	30 (100)	9 (30)	5 (16.7)			
	Unossified	0 (0)	21 (70)	25 (83.3)	0.00001	0.00001	*0.3604*
Sternum	Complete	23 (76.7)	0 (0)	0 (0)			
	Partial	7 (23.3)	21 (70)	10 (33.3)	0.00001	0	*1*
	Unossified	0 (0)	9 (30)	20 (66.7)	0.00001	0.00001	*1*
Ribs	Complete	30 (100)	19 (63.4)	6 (20)			
	Partial	0 (0)	9 (30)	3 (10)	0.0006	0	*1*
	Unossified	0 (0)	2 (6.6)	21 (70)	*0.1647*	0.00001	0.00001
Humerus	Complete	30 (100)	17 (56.7)	0 (0)			
	Unossified	0 (0)	13 (43.3)	30 (100)	0.00001	0.00001	0.00001
Radius and ulna	Complete	30 (100)	7 (23.4)	0 (0)		0.00001	
	Unossified	0 (0)	23 (76.6)	30 (100)	0.00001		0.0105
Metacarpals	Complete	17 (56.7)	3 (10)	0 (0)			
	Unossified	13 (43.3)	27 (90)	30 (1000	0.0003	0.00001	*0.2373*
Femur	Complete	30 (100)	18 (60)	0 (0)			
	Unossified	0 (0)	12 (40)	30 (100)	0.0001	0.00001	0.00001
Tibia and fibula	Complete	30 (100)	6 (20)	0 (0)			
	Unossified	0 (0)	24 (80)	30 (100)	0.00001	0.00001	0.0237
Metatarsals	Complete	23 (76.7)	4 (13.3)	0 (0)			
	Unossified	7 (23.3)	26 (86.7)	30 (100)	0.00001	0.00001	0.1124

Skeletal area	Control	Low dose	High dose	Fisher exact test			
	Number (%)	Number (%)	Number (%)	Value^1^	Value^2^	Value^3^	
(B)							
Dumbbell vertebrae	0 (0)	12 (40)	19 (63.33)				
Non Dumbbell vertebrae	30 (100)	18 (60)	11 (36.67)	0.0001	0.00001	0.1205	
Curved ribs	0 (0)	2 (6.67)	11 (36.67)				
Non curved ribs	30 (100)	28 (93.33)	19 (63.33)	*0.4915*	0.0003	0.0102	
Normal fore limb	30 (100)	30 (100)	13 (43.33)				
Abnormal fore limb	0 (0)	0 (0)	17 (56.67)	*1*	*0*	*0*	
Normal hind limb	30 (100)	30 (100)	11 (36.67)				
Abnormal hind limb	0 (0)	0 (0)	19 (63.33)	*1*	0.0003	0.0003	

Number of examined fetuses (n) = 30.

Fisher exact test statistic value^1^: between control and low dose. value^2^: between control and high dose. value^3^: between low dose and high dose. The *Italic* value is not significant at *P* < .05.

Fetuses of the LD group showed several variations; decreased degree of ossification in the bones of the median dorsal series (nasal, frontal, parietal, and inter-parietal) (Fig. 3B), incomplete ossification of the last rib, unossified lumbar vertebral centra (Figs. 4B,C), and incomplete ossification of the sternum (Fig. 5B). Absence of ossification of arm bones, such as the radius, ulna, metacarpals, and phalanges, also appeared as a result of low-dose treatment (Fig. 6B). The only skeletal malformation observed was the dumbbell-shaped thoracic vertebra (Figs. 4B,D). The fetuses of the HD group exhibited multiple variations; completely unossified skulls (Fig. 3C), vertebral centra (Fig. 4D,E), ribs (Fig. 4E), sternal bones (Fig. 5C), pectoral girdle, forelimb (Fig. 6C), and pelvic girdle (Fig. 7C). Additionally, exposure to high-dose lufenuron resulted in skeletal malformations; an abnormal increase in the curvature of the last rib (Fig. 4E), deformation of the radius, ulna (Fig. 6C), and hind limbs (Fig. 7C). The skeletal abnormalities were examined in 30 fetuses from each group and the percentage of the frequency and its significance Fisher’s exact test of each abnormality are shown in Table 3.

### Histopathologic examination

- Maternal liver tissue

**Figure 8:**
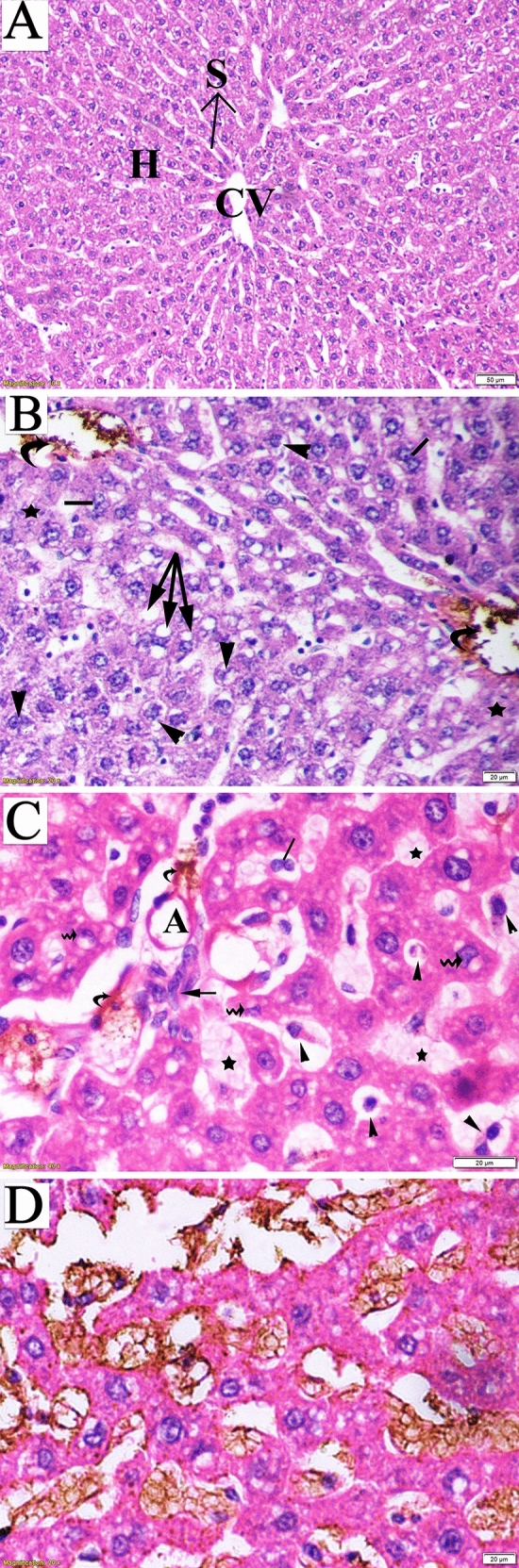
Photomicrograph of liver sections of pregnant dams (stained with hematoxylin and eosin) obtained on the 20th day of gestation. (**A**) Control group: normal lobular architecture with regular arrangement of hepatocytes (H) around a central vein (CV). Hepatocytes and their nuclei and blood sinusoids (S) were normal in shape. (**B**) Low-dose group: loss of hepatocyte architecture, damaged hepatocyte region (star), ballooning (head arrow), and binucleation (lines). Cellular vacuolization due to mild steatosis (arrow) and yellowish-brown pigment due to bile accumulation in portal space were also notable (curved arrow). (**C**) High-dose group: apoptotic cells (head arrow), crescentic nuclei of damaged hepatocytes (wavy arrow), and loss of hepatic artery endothelium (A) and disrupted bile duct (arrow) with bile accumulation in the portal space (curved arrow). (**D**) High-dose group: accumulation of golden brown finely granular lipofuscin pigment.

The hepatic architecture of the liver tissues obtained from the control group revealed a standard lobular structure with tightly packed hepatic cords radiating from the central vein to the periphery. The hepatocytes were regular in shape, showing large polygonal cells with large rounded nuclei. The hepatic strands were separated by a system of sinusoids that converged toward the central vein with normal dilation (Fig. 8A). The liver sections obtained from dams of the LD group exhibited intra-cytoplasmic fat droplets (mild steatosis) that appeared as cellular vacuolization in addition to damaged hepatocyte areas. Induced necrotic changes in the hepatocytes included ballooning degeneration characterized by vacuolated cytoplasm with shrunken and partially lysed nuclei. Destruction in the lobular structure and accumulation of bile secretions in the portal region (represented by yellowish-brown pigment) were also detected during the examination of the liver sections (Fig. 8B). In the liver tissue of the dams in the HD group, lobular structure showed notable lesions comprising the disorganized hepatic architecture and abnormal portal space structures; disrupted bile duct without distinct lumen (mild intrahepatic cholangitis), damaged endothelial lining of the hepatic artery, several apoptotic and crescentic hepatocytes, and intra-sinusoidal necrosis with accumulation of golden brown finely granular lipofuscin pigment along the hepatic cords (Fig. 8C,D).

b. Fetal liver tissue

**Figure 9:**
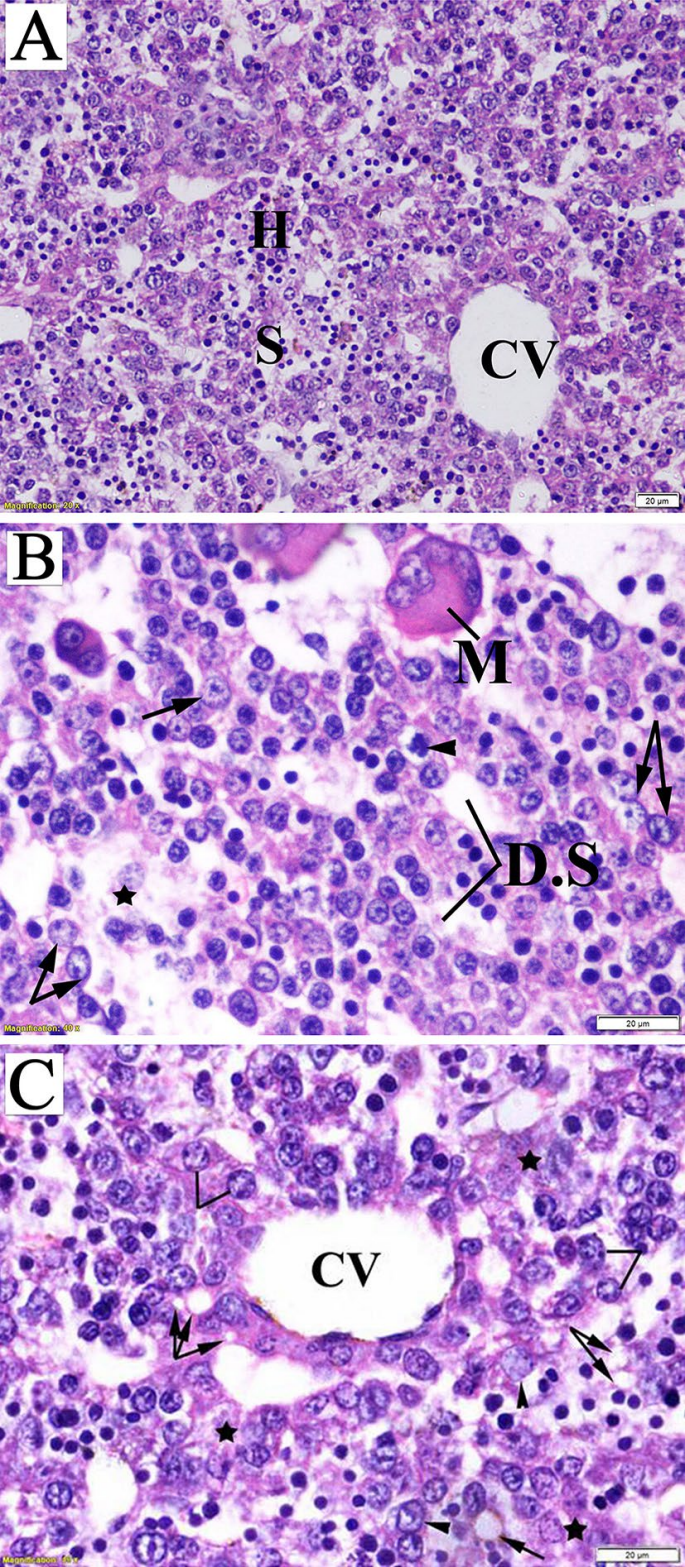
Photomicrograph of fetal liver sections (stained with hematoxylin and eosin) obtained on the 20th day of gestation. (**A**) Control group: normal structure of liver with normal hepatocyte nuclei. (**B**) Low-dose group: megakaryocyte (M), dilated sinusoids, ballooning degenerated cell (head arrow), and intracellular vacuolization with prominent nuclei (arrow) and area of complete loss of hepatic structure (star). (**C**) High-dose group: damaged endothelial lining of central vein (CV), intercellular vacuoles (arrow), damaged hepatocytes (star), and vacuolated hepatocytes (head arrow, lines).

The fetal liver tissue sections obtained from the control group were characterized by indistinctly marked hepatic lobules and irregularly branched and interconnected hepatic strands running from the central vein toward the periphery. The hepatocytes were polygonal in shape, with distinct boundaries and spherical nuclei. Blood sinusoids were in the form of irregularly dilated vessels (Fig. 9A).

Hepatocyte hypertrophy with vacuolated appearance and vesiculated nuclei accompanied by damaged hepatocyte regions was observed upon microscopic examination of fetal liver sections from the LD group. Destruction of liver architecture and erosion of endothelial cells lining the central vein were also noticeable (Fig. 9B). The fetal liver tissues obtained from the HD group showed a marked loss of lobular architecture. Signs of degeneration, including the presence of intracellular vacuolization, ballooning of hepatic cells, and damaged hepatocyte regions, were observed (Fig. 9C).

c. Maternal kidney tissue

**Figure 10:**
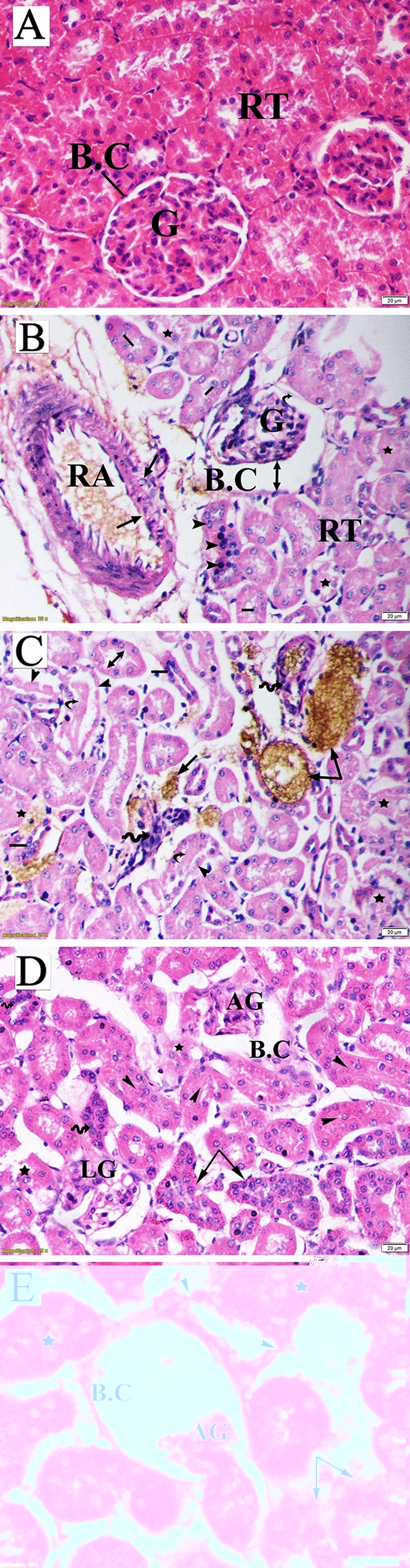
Photomicrograph of a transverse section of maternal kidney tissue (stained with hematoxylin and eosin) obtained on the 20th day of gestation. (**A**) Control group: normal histologic pattern of renal tissues; normal glomeruli (G) surrounded by Bowman's capsule (BC) and normal renal tubules (RT). (**B**) Low-dose group: shrunken glomeruli (G) surrounded by the deteriorated basement membrane of the BC (curved arrow), wide capsular space (double head arrow), damaged (star) and proliferating nuclei (head arrow) of tubular epithelium, shredded nuclei into the tubule lumen (lines), and dilation and damage of renal artery. (**C**) Low-dose group: calcium oxalate crystals (arrow), inflammatory infiltration (wavy arrow), tubular damage (star), protein (hyaline) cast deposition (curved arrow), dilation of the proximal tubule lumen (double head arrow), degenerated tubular epithelial lining (head arrow), and proliferating tubular cells (lines). (**D**) High-dose group: atrophied glomeruli (AG) surrounded by a fragmented capsule, lobulated glomeruli containing vacuoles, tubular degeneration (star), hypertrophy, and hyperplasia of the tubular epithelial lining (arrow). (**E**) High-dose group: BC with completely degenerated glomeruli, AG, deteriorated tubules (disconfiguration) (star), damaged tubules (arrow), and fragmented tubular epithelial lining (head arrow).

The examined renal sections of untreated pregnant rats showed normal structure. The renal corpuscle consisted of a glomerulus surrounded by the Bowman’s capsule with normal urinary space. The renal tubules included proximal convoluted tubules lined by pyramidal cells with an irregularly narrow lumen, and distal convoluted tubules with wider lumen lined by cuboidal epithelium without a brush border (Fig. 10A).

The kidney sections of mothers in the LD group revealed significant histopathologic changes comprised of shrunken and atrophied glomeruli, dilated urinary space, deteriorated basement membrane of the Bowman's capsule, damaged and proliferating nuclei of the renal tubule epithelium, sloughing of several cells into the lumen, collapsed tubules, and dilated lumen of proximal tubule. Furthermore, the renal sections of this group showed localization of calcium oxalate crystals inside damaged tubules with inflammatory infiltration, protein (hyaline) casts, and damaged renal artery (Fig. 10B,C).

In the renal tissue sections of the HD group, renal corpuscles showed varied degrees of glomerular alteration, including atrophy, lobulated with vacuoles, and completely damaged ones, along with eroded basement membrane of the Bowman’s capsule. Moreover, the renal tubules revealed different disconfigured patterns represented as complete or partial loss of tubular epithelium, hyperplastic tubular cells, narrowing of the lumen, collapsed tubules, and protein cast deposition (Fig. 10D,E).

d. Fetal kidney tissue

**Figure 11:**
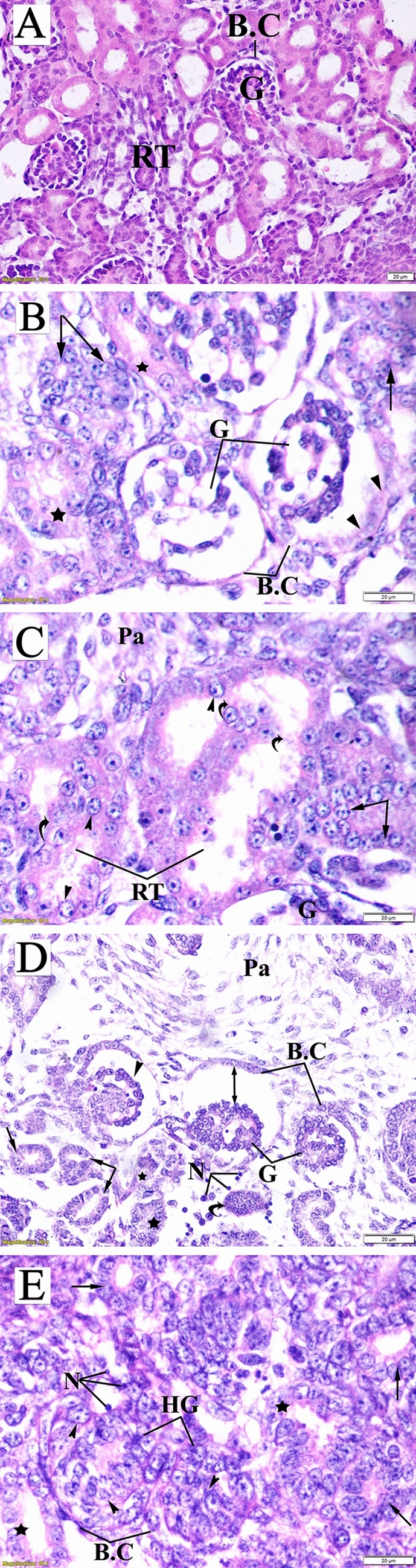
Photomicrograph of fetal kidney transverse sections (stained with hematoxylin and eosin) obtained on the 20th day of gestation. (**A**) Control group: distinct Bowman’s capsule (BC)-enclosed normal glomeruli (G) with normal urinary space. Renal tubules (RT) with normal nuclei were observed. (**B**) Low-dose group: damaged glomeruli (G) surrounded by a decomposed Bowman’s capsule basement membrane (head arrow), tubular cells karyolysis (arrow), and damaged tubules (star). (**C**) Low-dose group: Confluent bulky damage of RT, curved arrow) with ballooning degeneration of tubular epithelia (head arrow), karyolysis (arrow), and increase in the number of parenchymal cells (Pa). (**D**) High-dose group: comma-shaped glomeruli (head arrow), congestion and shrinkage of glomeruli (G), increased capsular space (double head arrow), necrotic cells (N), shredded renal tubules (arrow), and completely damaged tubules (star). Also, a vestige of degenerative renal capsule (curved arrow) and expansion of inter-tubular parenchyma (Pa) were observed. (**E**) High-dose group: glomerular hypertrophy, including damaged parital and mesangial cells (head arrow), Bowman’s capsule contracture (BC), necrosis (N), fragmented tubules (star), and ballooning degeneration of tubular epithelial cells (arrow).

Histologic examination of fetal kidney tissue of the control group exhibited normal tissue structure characterized by distinct Bowman’s capsule enclosing the glomerulus with normal cells and capillaries, as well as normal tubular structure and interstitial parenchyma (Fig. 11A).

The fetal kidney tissues obtained from the LD group exhibited a marked distortion of renal configuration, fragmented glomeruli, dissolution of the capsular basement membrane, massive damage of confluent renal tubular epithelia, karyolysis and ballooning degeneration of tubular epithelium nuclei, and mild increase of parenchymal cells (Fig. 11B,C).

In the HD group, fetal kidney sections showed degenerative renal tubular changes represented by ruptured fragmented tubules and foamy appearance of many tubular cells due to vacuolized degeneration. Several glomeruli appeared hypertrophied, with damaged mesangial and parietal cells and obliterated Bowman’s space. Additionally, shrunken, congested, and comma-shaped glomeruli were observed. Moreover, an expansion of the intertubular parenchyma and several necrotic cells were also detected (Fig. 11C,D).

### Assessment of DNA fragmentation by comet assay [single-cell gel electrophoresis (SCGE)]

**Figure 12:**
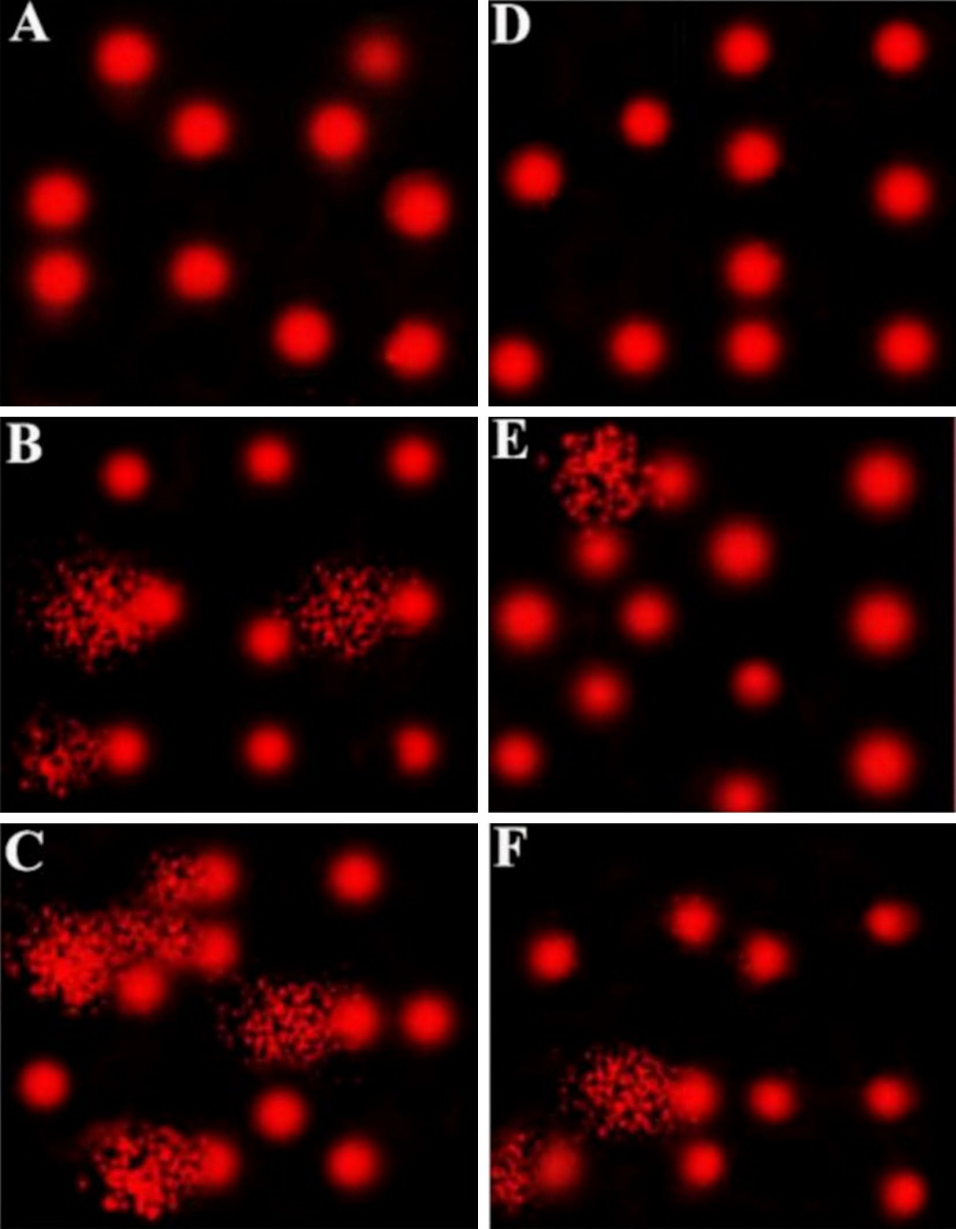
Photomicrographs that represent DNA damage in liver tissues detected through comet assay. (**A**) Control group maternal cells, (**B**) low-dose group maternal cells, (**C**) high-dose group maternal cells, (**D**) control group fetal cells, (**E**) low-dose group fetal cells, and (**F**) high-dose group fetal cells.

Comet assay was performed to assess DNA damage in maternal and fetal liver cells of lufenuron-exposed rats, compared with the control group. Exposure to both low and high doses led to a significant increase in DNA damage (*P* < 0.05) in the liver cells of both mothers and fetuses, which was indicated by increased tail length, tail DNA%, and tail moment, relative to the control group (Table 4). Analysis of the comet assay results showed a significantly increased tail length and tail moment readings (4.82 and 18.99, respectively) in the LD group, relative to the control group (1.72 and 2.23, respectively). The increase in tail length and moment (9.75 and 84.60, respectively) in the liver cells obtained from the HD group was highly significant, implying the massive DNA damage caused by exposure to lufenuron. The same effect was observed in the fetal liver cells, although at a lesser extent. Taken together, exposure to lufenuron resulted in significant DNA damage in liver cells of both mothers and fetuses of the treatment groups in a concentration-dependent manner (Fig. 12).

**Table 4:** Comet assay parameters obtained by image analysis of liver cells obtained from the three groups.

Group/parameter	Tailed (%)	Untailed (%)	Tails length (µm)	Tail DNA (%)	Tail moment
Maternal control	3	97	1.72 ± 0.12	1.30	2.23
Maternal LD	15	85	4.82 ± 0.19*	4.11	18.99
Maternal HD	28	72	9.75 ± 0.62**	8.66	84.60
Fetal control	2	98	1.44 ± 0.13	1.51	2.16
Fetal LD	6.5	93.5	2.35 ± 0.20*	2.69	6.32
Fetal HD	12	88	4.58 ± 0.15**	3.67	16.81

*,**Significant difference (*P* < 0.05), as compared to the control group.

### Cell cycle analysis through flow cytometry using propidium iodide (PI) staining

**Figure 13:**
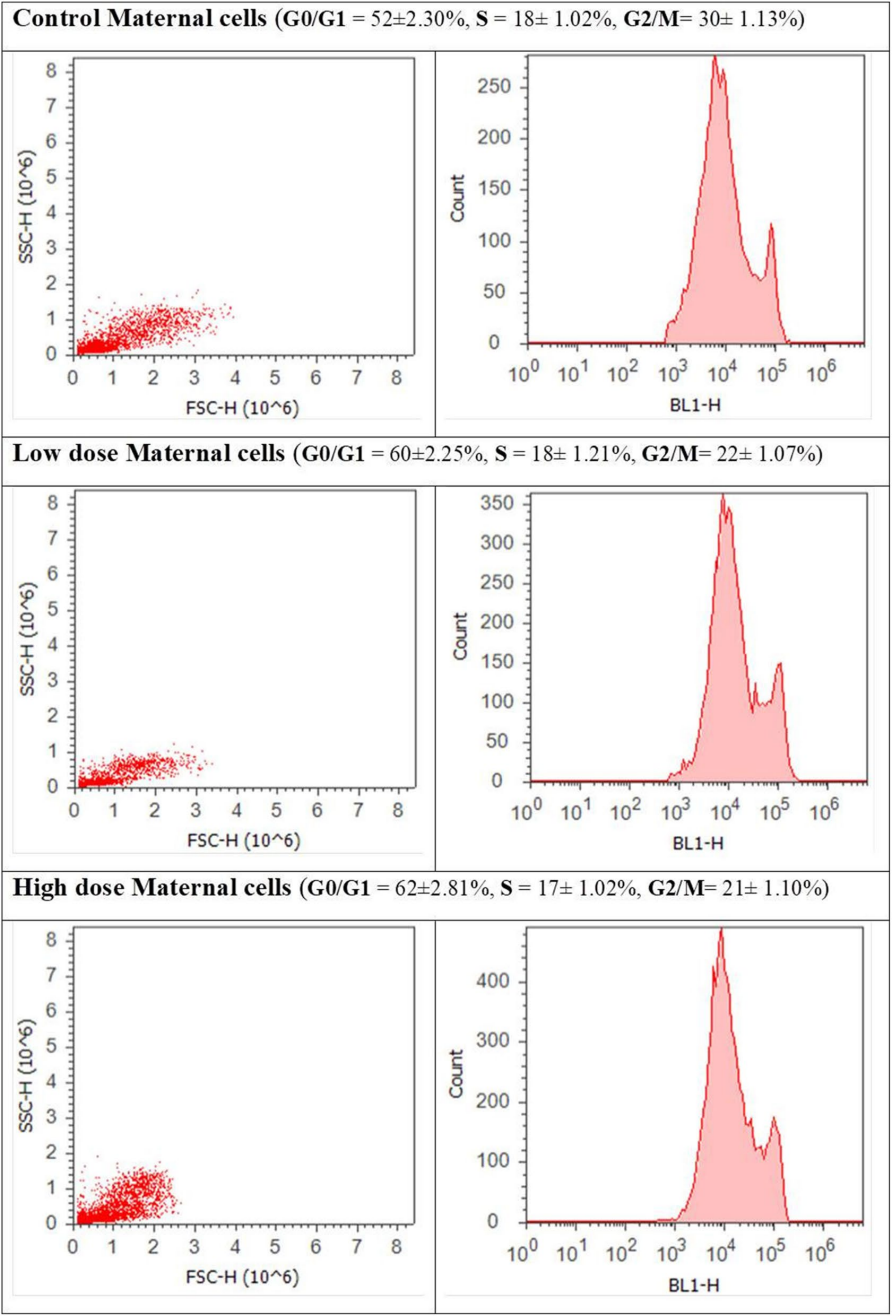
Cell cycle analysis of maternal liver cells of the three groups (control, low-dose, and high-dose groups) through flow cytometry using propidium iodide staining.

**Figure 14:**
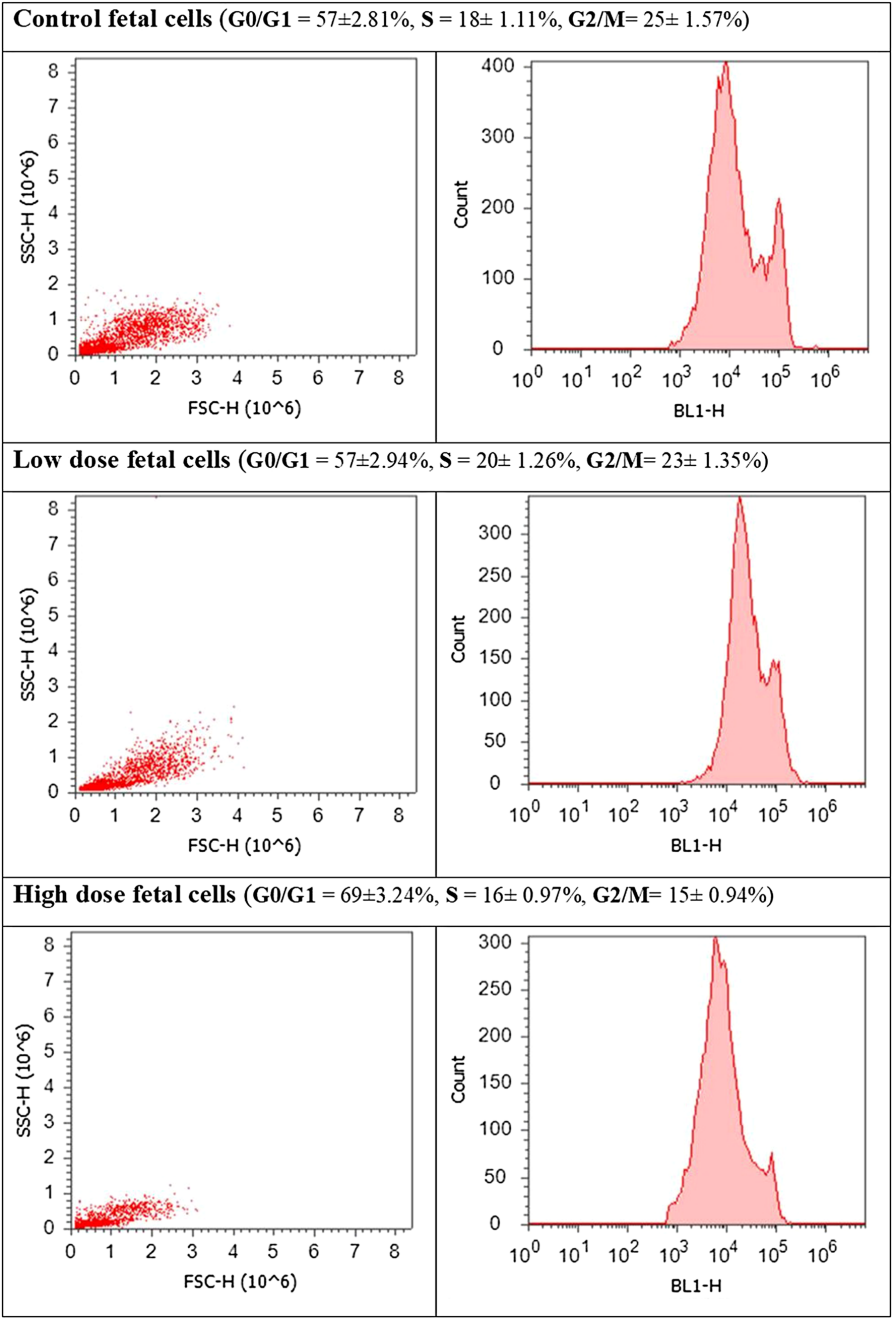
Cell cycle analysis of fetal liver cells of the three groups (control, low-dose, and high-dose groups) through flow cytometry using propidium iodide staining.

Cell cycle analysis for liver cells obtained from both mothers and fetuses of the treatment groups revealed cell cycle perturbation in both of them. Maternal cells obtained from the LD and HD groups showed a significant increase of cell percentage in the G0/G1 phase and a clear decrease in the G2/M phase, relative to the control group. There was no significant effect in the S phase (Fig. 13). Fetal liver cells obtained from the LD group showed a slightly significant increase in the S phase accompanied by an insignificant decrease in cell percentage in the G2/M phase. The HD group’s fetal cells showed a highly significant increase in cell percentage in the G0/G1 phase, relative to that of the control group. An insignificant decrease was recorded in cell percentage in the S phase, whereas that in the G2/M was significantly decreased (Fig. 14).

### Estimation of oxidative stress

**Figure 15:**
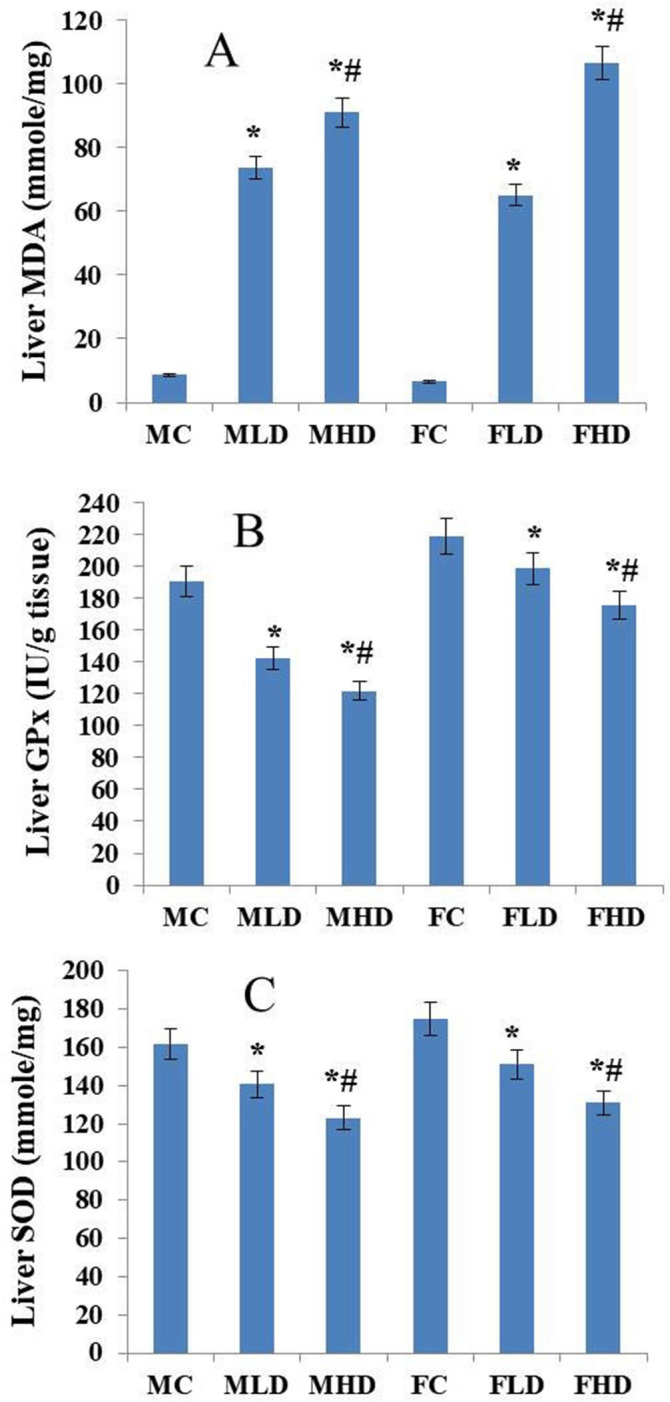
Histogram representing the estimation of oxidative stress markers. (**A**) malondialdehyde (MDA) level in liver cells of mothers and fetuses of the three groups based on the MDA spectrophotometric assay. (**B**) glutathione peroxidase activity (GPx) in liver cells of mothers and fetuses of the three groups based on the GPx spectrophotometric assay. (**C**) Superoxide dismutase activity (SOD) in liver cells of mothers and fetuses of the three groups based on the SOD spectrophotometric assay. *MC = maternal control, MLD = maternal low dose, MHD = maternal high-dose, FC = fetal control, FLD = fetal low dose, and FHD = fetal high-dose. Histograms were generated using Microsoft Excel software.

Analysis of data collected from the spectrophotometric assay for liver cells obtained from mothers and fetuses of the three groups revealed the potential of lufenuron treatment for posing oxidative stress on liver cells of the pregnant dams and their fetuses in a dose-dependent manner. The maternal liver MDA level in the LD (73.76 ± 1.13) and HD (91.10 ± 1.65) groups was found to be significantly higher (*P* < 0.05) than that of the control group (8.61 ± 2.12). A similar effect was observed in the fetal liver samples, where in the MDA level was significantly increased in both the LD (65.18 ± 0.54) and HD (106.71 ± 0.75) groups, relative to that of the control group (6.52 ± 3.91) (Fig. 15A). The hepatic GPx and superoxide dismutase (SOD) activities were decreased in both the mothers and fetuses of the treatment groups. In the maternal liver, when compared with the control group (190.96 ± 5.81), GPx activity was lower (142.10 ± 5.31 and 121.94 ± 5.72 IU/g tissue) in the LD and HD groups, respectively. In the fetal liver, GPx activity was also decreased in the LD (198.96 ± 4.22) and HD (176.11 ± 4.65) groups, relative to the control group (219.04 ± 6.47) (Fig. 15B). Hepatic SOD activity was also decreased in mothers and fetuses of the LD (140.82 ± 3.12 and 151.12 ± 2.01, respectively) and HD (123.16 ± 3.45 and 130.87 ± 2.11, respectively), relative to the control group (161.51 ± 4.32 and 174.66 ± 5.02, respectively) (Fig. 15C).

## Discussion

In developing countries, ignorance of safe management protocols, along with excessive and widespread use of insecticides and other agrochemicals, have resulted in contamination of various compartments of the environment and exposure of the general population to the harmful effects of these chemicals^46–48^. What deepens the problem is that most of the chemicals that are used as pesticides are not necessarily selective; however, they may be generally toxic to many non-target species, including man, and other lifeforms^49^. The controversy that is generated regarding the excessive use of chemical pesticides leads to the evolution of more biologically harmful pesticides, such as IGRs, including lufenuron that was previously considered to be safe^50,51^.

One of the major concerns about the consequences of pesticide residue exposure is the transmission of exposure-induced damage to the next generations. Several studies suggest an association between the environmental exposure of pregnant women to certain agricultural pesticides and malformations in their fetuses^52^. In the present study, exposure to lufenuron at concentrations 0.4 and 0.8 mg/kg resulted in reduction in the maternal body weight gain but no deaths were recorded among the pregnant females. The decreased body weight might be attributed to the direct effect of lufenuron or might be a palatability effect of oral administration of lufenuron that led to lower appetite. In a recent study, Shahid and Saher^53^ documented a significant less body weight gain in the pregnant mice exposed to pyriproxyfen. The present study also showed a significant reduction in the uterine horn dimensions and weight which might be due to the reduced number of live fetuses and the increased percentage of resorption in the two treated groups compared to control. Previous studies showed that exposure of mice and rats to mancozeb, methyl parathion, methoxychlor, heptachlor, and chlordimeform may lead to a decrease in uterus weight as well, which may affect implantation^54–58^. Lufenuron treatment induced fetal growth retardation and developmental disorders, including hematoma, total foot loss, skeletal malformation (Dumbbell-vertebrae, Curved ribs, and deformation of the radius, ulna and hindlimbs), and death. Several previous studies have reported limb reduction defects as a result of exposure to insecticides^59,60^. Delayed or incomplete ossification of fetal bones may be related to the effect of the insecticide on calcium metabolism or deviations in calcitonin levels in developing fetuses, leading weak bone development^61^. The reduced ossification of fetal skeletons may reasonably explain the decrease in fetal weight. A similar relationship between reduced fetal body weight and retarded skeletal ossification has been suggested by Murray et al.^62^ following Carbaryl exposure of rabbits and mice. In agreement with our results, lufenuron was found to induce structural and skeletal anomalies in developing embryos when injected in pre-incubated eggs of *Gallus domestics*^45^. Teratogenic development of chick embryo upon exposure of the mother to chlorantraniliprole, chlorpyrifos, cypermethrin, endosulfan, malathion and spinosad was also reported^63–67^. Moreover, increased fetal resorption and reduced number of live fetuses, along with skeletal abnormalities, were reported as signs of teratogenicity for fetuses of pregnant rats exposed on gestation day 6 through day 15 to chlorpyrifos^68^, cypermethrin^69,70^, dimethoate^69,71^, endosulfan^72^, emamectin benzoate^73^, fipronil^74^, and organophosphorus insecticides^75^.

In the present study, both of the tested lufenuron concentrations were found to cause several histopathologic changes in liver and kidney tissues obtained from both lufenuron-treated dams and their fetuses. Histologic examination revealed intrahepatic cholangitis and steatosis in maternal liver tissues along with necrotic damage in the tissue of the mothers and fetuses. Maternal renal tissues showed damaged and proliferating renal tubular epithelia, damaged glomeruli, and accumulation of calcium oxalate crystals with inflammatory infiltration. In fetal kidney tissues, lufenuron distorted the renal configuration, deteriorated the Bowman’s capsule, caused massive damage to renal tubular epithelia, and increased the number of intratubular parenchyma. In the same vein, lufenuron, at residual concentrations, was found to induce histopathologic changes in the livers of albino mice^76^. The same histopathologic effects were discovered in the liver and kidney tissues of rats after administration of lufenuron even after the recovery period^77^. Rather similar results were obtained in rats treated with other insecticides, such as fipronil^78^ and dursban^79^. Histopathologic changes were also recorded in the kidney tissues of mice after exposure to the IGR pyriproxyfen^80^.

The genotoxic potential of these chemicals is probably the major risk factor in long-term effects, such as carcinogenic and degenerative diseases^81^. In the present study, both of the tested lufenuron concentrations induced a high level of DNA damage along with cell cycle perturbations, which suggests the high genotoxic potential of this insecticide. Previous studies have proven lufenuron to be a potent mutagen to both germ-line and somatic cells of *D. melanogaster*^82^. It was also proven to interfere with the spindle fiber formation of mice spermatid cells^76^ and induce micro as well as macro genetic lesions in the mice genome^83^. At the molecular level, significant DNA damage was recorded through a comet assay in *D. melanogaster*^34^ and *Biomphalaria alexandrina*^84^ after exposure to lufenuron. The same results were recorded for isolated mice peripheral blood lymphocytes after exposure to carbofuran^85^.

Cell cycle perturbation is considered as a major feature of apoptosis^86^. PI staining and flow cytometry are the most commonly used methods to quantitate DNA content in different cell cycle phases. Moreover, PI is an intercalating agent that is impermeable to live cells; hence, it can distinguish dead cells from live cells^87^. Several previous studies have discussed the effect of pesticides on cell cycle arrest. Cell cycle perturbations were detected in human peripheral blood mononuclear cells and adenocarcinoma human alveolar basal epithelial (A549) cell lines exposed to atrazine, butachlor, chlorpyriphos, and dichlorvos^88^; and bone marrow cells of Swiss albino mice exposed to formulated cypermethrin and/or chlorpyrifos^89^. Cypermethrin, one of the most highly effective synthetic pyrethroid insecticides, induced G1 cell cycle arrest associated with an enhanced expression of p21, wild-type p53, and down-regulation of cyclin D1, cyclin E and CDK4 in RAW 264.7 cells in a dose-dependent manner^90^. Thiacloprid-based insecticide treatment resulted in decreased cell viability and proliferation, p53-mediated cell cycle arrest at the G_0_/G_1_ phase, and apoptosis induction accompanied by elevated levels of mitochondrial superoxide and protein carbonylationin bovine lymphocytes^91^. In a more recent study, in response to exposure to a pesticide mixture of imidacloprid and iprodione, the cell cycle of *Allium cepa* root was blocked in the G_1_ phase^92^.

Oxidant-mediated responses, such as apoptotic or necrotic cell death, membrane lipid peroxidation, metabolic perturbation, and deregulation of several signaling pathways) are several well-known toxicological effects of insecticides^93,94^. Hence, the study of antioxidant enzymes has been used as a popular method to evaluate the response of multiple animal models, including rats, mice, fish, and green snake head, under pesticide-induced stress conditions^78,95–100^. In the present study, lufenuron was found to increase the level of MDA and decrease the level of GPx and SOD enzymes in liver cells in mothers and their fetuses in a dose-dependent manner, which refers to the oxidative stress posed by the insecticide on the liver cells of the treated animals. Deivanayagam et al.^101^ reported that the levels of lipid peroxidation were increased and that of GPx, catalase (CAT), and superoxide dismutase were significantly decreased in the liver tissue of mice exposed to a sublethal dose of lufenuron (0.1520 mg/kg). Pesticides may directly inhibit GPx by impairing the functional groups, or indirectly by rendering the supply of reduced glutathione and NADPH. Sharma et al.^102^ reported that the decreased level of antioxidant defense system is the main factor responsible for generating hydroxyl radicals that promote oxidative damage by Fenton reaction. Inhibition of antioxidant defense may be coupled with the depletion of glutathione^103^. Oxidative stress biomarkers such as SOD, CAT, and GPx were also found to be significantly decreased in fipronil- and glyphosate-treated rats^78,99^ as well as in Diazinon treated mice^104^. Triflumuron increased MDA levels in human colon carcinoma cells HCT116 cell lines and mice^105,106^. Similar results were reported in isolated rat hepatocytes treated with fenitrothion, endosulfan, and abamectin^107^.

## Conclusion

The results of the assessment of reproductive teratogenicity of lufenuron, morphologic examination of fetuses, and staining for skeletal malformations showed the tested concentrations of lufenuron can induce teratogenic effects on the fetuses of rats if the exposure occurred during the organogenesis period. The results of the histopathologic examination of liver and renal tissues of both mothers and fetuses showed that the tested lufenuron concentrations can cause histopathologic changes in the examined tissues. The results obtained from the comet assay, flow cytometry, and the assays for estimation of oxidative stress (MDA, GPx and SOD), relative to those in the control group, have proven the ability of both tested concentrations of lufenuron to induce genotoxic stress, cell cycle arrest, and oxidative stress, respectively. These results on rats imply that women and their fetuses may be exposed to the same risk. Further investigation needs to be conducted on rats in the 3rd gestational period.

## Materials and methods

### Tested chemical

The chemical used in this study, Lufenuron, is an acylurea insecticide with the chemical formula(1-[2,5-dichloro-4-(1,1,2,3,3,3-hexafluoropropoxy)phenyl]-3-(2,6difluorobenzoyl) urea). 5% emulsifiable concentrate was purchased from Syngenta Egypt under the commercial name match 5%. Doses were prepared shortly before administration by dissolving lufenuron in distilled water to achieve the desired concentration in 1 ml of the solution.

### Experimental animals

Animals used in this study were healthy (checked by vet for common problems as obesity and breathing problems and were also observed closely for signs of stress, pain, illness and injury) Sprague–Dawley rats. Males and virgin females, weighing between 140 to 180 g were procured from the animal house of the National Research Center, Egypt. All animals were allowed to acclimate in a laboratory environment with free access to food and water for one week before the onset of the experiment. Light–dark photoperiod was maintained at 12:12 h in a controlled environment temperature of 22 ± 1 °C and relative humidity of 55 ± 5%^108^. All experiments and procedures were performed in accordance with relevant guidelines/regulations of ethical committee. The experiments were consented by The Institutional Animal Care and Use Committee (IACUC), Faculty of science, Cairo University with the approval number (CU/I//F/29/18).

### Crosses and treatments

Every two females were housed overnight with one male and left for mating. Females were under daily scrutiny for the vaginal plug (a white plug fills most of the vaginal cervical junction) and preparation of vaginal smear which signifies the first day of gestation according to Burdan et al.^109^. Pregnant females were caged separately and classified randomly into three groups, each containing ten females.

Both of the control and treated groups were orally administrated with the corresponding treatment of distilled water and lufenuron, respectively, once daily at the same time over the seven days of the second gestation period (from 7 to 13th gestational day) as follows:

- Control group: Pregnant rats received an equivalent volume of vehicle (distilled water) by gavage.
- Low dose group (LD): Pregnant rats were treated with a low dose of lufenuron (0.4 mg/kg of body weight) by gavage.
- High dose group (HD): Pregnant rats were treated with a high dose of lufenuron (0.8 mg/kg of body weight) by gavage.

The doses under investigation were selected based on the maximum residual levels (MRL) in some crops according to European Food Safety Authority (EFSA) report^110^. All dams of treated groups were observed daily for any signs of general toxicity, bleeding, and/or mortality. Body weight was recorded weekly throughout gestation.

### Assessment of reproductive teratogenicity of lufenuron

On the 20th gestational day, the dams were euthanized using sodium pentobarbital and subjected to cesarean section operations. The ovaries of both uterine horns were removed, and the corpora lutea were counted. Gravid uteri were carefully segregated, weighed, and examined. The total number of implantation sites, as well as live, resorbed, or dead fetuses, was recorded according to a previously described method^111,112^.

The post-implantation loss index was calculated^111^ as follows:

$$\begin{aligned}\%\; of \;post{\text{-}}implantation \;loss &=\frac{Number\; of \;implantation \;site-Number \;of \;live\;fetuses}{Number\;of\; implantation \;sites }\times 100\\ \% \;of \;resorption &=\frac{Number \;of \;resorption \;sites}{Number\;of \;implantation \;sites }\times 100 \end{aligned}$$

The fetuses were freed from their membranes, separated from their placentae, examined for any morphologic malformations, and then the body weight and crown-rump length values of each fetus were recorded^113^.

### Examination of fetal skeleton for abnormalities

Fetuses were fixed in 100% ethanol, then eviscerated and stained using Alcian blue–Alizarin red double staining method according to Young et al.^114^. The stained fetal skeleton was stored in pure glycerol to be examined for any anomalies according to Aliverti et al.^115^ using a dissecting binocular microscope (Leica, Germany) and photographed using a digital camera (Leica, Germany).

### Histopathologic examination

Tissue biopsies from the liver and kidney of both mothers and fetuses from all three groups were immediately taken and fixed using 10% neutral-buffered formalin for 24 h. Preparation of paraffin sections and staining in hematoxylin and eosin was performed according to the method previously described by Bancroft et al.^116^. All sections were examined using a BX53M light microscope (Olympus, Japan) and photographed using a DP74 camera (Olympus, Japan).

### Assessment of DNA fragmentation by comet assay (SCGE)

Liver tissues were frozen in liquid nitrogen and gently homogenized into a powder. The extent of DNA strand breaks in the liver tissues of mothers and fetuses from the three groups was assessed using the alkaline comet assay, as previously described by Tice et al.^117^. Comets were analyzed using an Axio fluorescence microscope (Carl Zeiss, Germany) with an excitation filter at 524 nm and a barrier filter at 605 nm. A Komet 5.0 analysis system developed by Kinetic Imaging, Ltd. (Liverpool, United Kingdom) connected to a charge-coupled device (CCD) camera was used to measure tail length, percentage of migrated DNA, and tail moment.

### Cell cycle analysis by flow cytometry through PI staining

Liver tissues acquired from the mothers and fetuses from all groups were weighed, rinsed in phosphate-buffered saline, and minced using a pair of scissors to produce approximately 1-mm fragments. Afterward, the tissues were digested using collagenase (1 ml/0.25 g tissue)^118,119^. Cell cycle analysis through PI staining was performed according to the method previously described by Allen and Davies^120^. The stained cells were analyzed using an Attune flow cytometer (Applied Biosystem, USA).

### Estimation of oxidative stress markers

The appropriate kits for determining malondialdehyde (MDA), glutathione peroxidase (GPX), and super oxide dismutase (SOD) were purchased from (Biodiagnostic, Egypt). Hepatic activities for MDA, GPX, and SOD were measured using a UV-2100 spectrophotometer (Qualitest, USA) according to Ohkawa et al.^121^, Paglia and Valentine^122^, and Nishikimi et al.^123^, respectively.

### Statistical analysis

Statistical analysis was performed using the SPSS software version 22. One-way analysis of variance was used to study the effects of both treatment types on the studied parameters. Post-hoc Duncan’s multiple range test was conducted to study the similarities in the studied variables among the experimental groups.
